# Supplementary material for: Improving Cancer Diagnosis in Alberta, Canada: A Qualitative Study of Emergency Department Healthcare Providers’ Perspectives on Diagnosing Cancer in the Emergency Setting
Source: Curr Oncol. 2024 Dec 25;32(1):5. doi: 10.3390/curroncol32010005 (PMC11764333; doi:10.3390/curroncol32010005)
Supplement: Supplementary file 1 [file curroncol-32-00005-s001.zip › curroncol-3368439-supplementary.pdf]

Table S1. Themes and subthemes from ED providers' perspectives on ED cancer diagnosis.

| Themes | Subthemes |
| --- | --- |
| 1. ED teams focus on acute presentations | - ED's primary role: Addressing acute symptoms and immediate stabilization<br>- Cancer diagnosis often incidental to primary presentation<br>- Transitioning patients to other settings for follow-up care<br>- Evolving responsibilities: Increased role in diagnostics and referrals due to limited access to primary care<br>- Challenges of balancing acute care focus with evolving diagnostic and care coordination responsibilities |
| 2. ED is not the ideal environment for cancer diagnosis | - Lack of privacy or appropriate spaces for sensitive conversations<br>- High-pressure, task-oriented workflows limiting time for patient interactions<br>- Limited continuity of care within the ED<br>- Lack of established patient-provider relationships<br>- Insufficient diagnostic and follow-up information available to ED providers<br>- Emotional challenges for patients in receiving and processing diagnosis<br>- Emotional burden on providers in delivering diagnoses and managing patient uncertainty |
| 3. Improved ED cancer diagnosis requires reducing patient volumes and optimizing diagnosis processes across the healthcare system | - Improving access to primary care to reduce unnecessary ED visits<br>- Expanding primary care capacity for timely testing and specialist referrals<br>- Raising public awareness on cancer prevention, symptoms, and screening<br>- Establishing a centralized intake and referral system<br>- Introducing dedicated personnel to support care coordination and patient support<br>- Streamlining diagnostic pathways to reduce delays in diagnosis and increase care continuity |
